# Impact of Docetaxel on blood-brain barrier function and formation of breast cancer brain metastases

**DOI:** 10.1186/s13046-019-1427-1

**Published:** 2019-10-29

**Authors:** Simon Bernatz, Elena I. Ilina, Kavi Devraj, Patrick N. Harter, Klaus Mueller, Sascha Kleber, Yannick Braun, Cornelia Penski, Christoph Renner, Rashi Halder, Lukas Jennewein, Christine Solbach, Frits Thorsen, Bernhard C. Pestalozzi, Axel Mischo, Michel Mittelbronn

**Affiliations:** 1 Edinger Institute, Institute of Neurology, University of Frankfurt am Main, Frankfurt, Germany; 2 Luxembourg Center of Neuropathology (LCNP), Luxembourg, Luxembourg; 3 Department of Oncology, Luxembourg Institute of Health (LIH), NORLUX Neuro-Oncology Laboratory, Luxembourg, Luxembourg; 4 Frankfurt Cancer Institute (FCI), Frankfurt am Main, Germany; 5 German Cancer Research Center (DKFZ), Heidelberg, Germany; 6 German Cancer Consortium (DKTK), Heidelberg, Germany; 7 Oncology Centre Hirslanden and Zurich, Zurich, Switzerland; 8 Luxembourg Centre for Systems Biomedicine (LCSB), University of Luxembourg, Esch-sur-Alzette, Luxembourg; 9 Department of Gynecology and Obstetrics, School of Medicine, J. W. Goethe-University, Theodor-Stern-Kai 7, D-60590 Frankfurt, Germany; 10 KG Jebsen Brain Tumor Research Centre, University of Bergen, Bergen, Norway; 11 Molecular Imaging Center, Department of Biomedicine, University of Bergen, Bergen, Norway; 12 Department of Medical Oncology and Hematology, University Hospital Zurich (USZ), Rämistrasse 100, CH-8891 Zurich, Switzerland; 13 National Center of Pathology (NCP), Luxembourg Center of Neuropathology (LCNP), Laboratoire national de santé (LNS), 1, Rue Louis Rech, L-3555 Dudelange, Luxembourg

**Keywords:** Brain metastasis, Docetaxel, Taxane, Breast cancer, BBB, TEER

## Abstract

**Background:**

Breast cancer (BC) is the most frequent malignant tumor in females and the 2nd most common cause of brain metastasis (BM), that are associated with a fatal prognosis. The increasing incidence from 10% up to 40% is due to more effective treatments of extracerebral sites with improved prognosis and increasing use of MRI in diagnostics. A frequently administered, potent chemotherapeutic group of drugs for BC treatment are taxanes usually used in the adjuvant and metastatic setting, which, however, have been suspected to be associated with a higher incidence of BM. The aim of our study was to experimentally analyze the impact of the taxane docetaxel (DTX) on brain metastasis formation, and to elucidate the underlying molecular mechanism.

**Methods:**

A monocentric patient cohort was analyzed to determine the association of taxane treatment and BM formation. To identify the specific impact of DTX, a murine brain metastatic model upon intracardial injection of breast cancer cells was conducted. To approach the functional mechanism, dynamic contrast-enhanced MRI and electron microscopy of mice as well as in-vitro transendothelial electrical resistance (TEER) and tracer permeability assays using brain endothelial cells (EC) were carried out. PCR-based, immunohistochemical and immunoblotting analyses with additional RNA sequencing of murine and human ECs were performed to explore the molecular mechanisms by DTX treatment.

**Results:**

Taxane treatment was associated with an increased rate of BM formation in the patient cohort and the murine metastatic model. Functional studies did not show unequivocal alterations of blood-brain barrier properties upon DTX treatment in-vivo, but in-vitro assays revealed a temporary DTX-related barrier disruption. We found disturbance of tubulin structure and upregulation of tight junction marker claudin-5 in ECs. Furthermore, upregulation of several members of the tubulin family and downregulation of tetraspanin-2 in both, murine and human ECs, was induced.

**Conclusion:**

In summary, a higher incidence of BM was associated with prior taxane treatment in both a patient cohort and a murine mouse model. We could identify tubulin family members and tetraspanin-2 as potential contributors for the destabilization of the blood-brain barrier. Further analyses are needed to decipher the exact role of those alterations on tumor metastatic processes in the brain.

## Background

Breast cancer (BC) is the most common cancer in females, and also the most frequent cause of cancer-related death in women in less developed countries, as well as the second cause in more developed regions after lung cancer [1]. In western countries it is not the primary tumour, but rather the metastatic disease, which is the main cause of death [2]. Approximately 20% [99/474] of women initially diagnosed with node-negative BC and 40% [67/170] with node-positive BC, develop recurrent or metastatic disease, when treated with radical mastectomy without chemotherapy [3]. In order to establish a distant metastasis, the tumor cells (TC) need to pass a series of sequential steps, known as the metastatic cascade: (i) invasion of adjacent tissue to intravasate in the circulatory system, (ii) arrest within the capillary bed, (iii) extravasation and finally (iv) proliferation at a secondary site [4–7]. Brain metastases (BM) are the most common intracranial tumors in adults being almost up to ten-fold more common than primary brain tumors and some primary tumors, among others BC, exhibit a particularly high BM incidence [8, 9]. For BM formation in the CNS microenvironment, TCs need to cross the blood-brain barrier (BBB) [10–12]*.* The BBB consists of ECs, lined by pericytes, basement membrane and astrocytes, forming a tight barrier around blood vessels [11, 12]. After passing the BBB, TCs can grow in the CNS, where they might potentially be protected from therapeutic agents [13]. Diagnosis of BM leads to a dismal prognosis with median overall survival of 13.8 months, ranging from 3.35 months to 25.3 months according to the specific Graded Prognostic Assessment Score [14]. Therefore, identification of possible risk factors, that lead to an increased amount of BM, are of high importance. The current treatment approaches for BM of BC patients are complex and numerous clinical trials are ongoing. Chemotherapeutic strategies often include members of the taxane family, leading to longer progression free- and overall survival [15, 16]. The traditional main agents of the taxane family, that are used in BC, are paclitaxel and DTX [17]. They act via permanent stabilization of assembled microtubules, thus impairing their dynamics and, consequently, cell mitosis and proliferation. Furthermore, taxanes induce apoptosis, however the underlying mechanisms are not yet fully understood [18, 19]. Controversial data exist regarding the frequency of CNS-relapse in patients treated with adjuvant taxanes, with some studies claiming the possibility of increased risk of BM formation upon taxane treatment [20–22]. Although taxanes are part of the standard treatment regime in BC, there is a lack of data concerning the impact of DTX treatment on BBB function and circulating TCs in the process of BM formation. The aim of this study was to assess the impact of DTX on BBB properties and formation of BM, using in-vitro and an in-vivo models. Furthermore, we aimed at characterizing the underlying mechanism.

## Methods

### Patient cohort and clinical data

Eighty breast cancer patients, treated in the Goethe-University hospital Frankfurt am Main, department of gynecology, from 2009 to 2015 were analyzed retrospectively as a case-control study. Patients reaching the primary end-point “brain-metastases” (BM, cases: *n* = 40) and patients without BM, but suffering from bone metastases (BoM, controls: n = 40), were reviewed for exposure to taxane-treatment prior to brain metastatic disease. Patient characteristics are summarized in Table 1. Statistical analysis was conducted using JMP 14 (SAS, Cary, U.S.A.), detailed description of the used statistical methodology is provided in the corresponding figure legend.

**Table 1:** Patient characteristics including treatment and pathological parameters

	BoM/ nBM (N)	BM (N)
patients	40	40
deceased	14	14
Taxane (#)	30	38
DTX	9	15
Paclitaxel	17	14
both	4	9
mean follow-up (##)		
days	2492.95	2545.15
years	6.83	6.97
age primary diagnosis (##) years	55.6	52.4
neoadjuvant therapy (#)		
yes	7	4
no	29	29
u	4	7
T (#)		
T1	9	7
T2	15	13
T3	5	7
T4	6	7
Tx	1	1
Tis	0	1
u	4	4
N (#)		
N0	10	16
N1	13	11
N2	5	4
N3	8	5
u	4	4
M (#)		
M0	17	23
M1	15	8
Mx	1	1
u	7	8
grading (#) *		
G1	4	0
G2	14	16
G3	9	17
u	13	7
ER (#) ***		
+	33	17
-	5	19
u	2	4
PR (#) *		
+	26	15
-	12	20
u	2	5
HER2/neu (#) *		
+	13	12
-	24	21
u	3	7
triple negative (#) *		
yes	3	9
no	34	27
u	3	4
intrinsic subtype (#) ***		
luminal	34	19
basal-like	3	9
Erb-B2 overexpression	0	8
u	3	4

*BoM* bone metastasis, *nBM* no brain metastasis, *BM* brain metastasis, *u* unknown. Statistical analysis was conducted using contingency analysis with likelihood-ratio/ Pearson test (#) or analysis of variance using one-way ANOVA (##). Significant differences are depicted as followed: * *p*<0.05; ** *p*<0.01; *** *p*<0.001

### Cell culture

The following cell types were used in our study: primary mouse brain microvascular endothelial cells (MBMEC), primary human brain microvascular endothelial cells (HBMEC), murine immortalized brain endothelial cells (bEnd5, obtained as described previously [23]) and human brain seeking breast-cancer cells MDA-MB-231-BR-GFP (BR231, kind gift from Dr. P. Steeg, National Cancer Institute, Bethesda MD 20892). ECs were cultured on 0.1% gelatin-coated flasks/dishes in MCDB-131 complete medium as previously described [24], BR231 cells were cultured in complete medium (DMEM+GlutaMAX, 10%FCS, 100 U/mL penicillin, 100 μg/mL streptomycin) in a humidified incubator. bEnd5 cells were cultured as described previously [24] and used in order to reduce the amount of animals required for generation of primary MBMECs. All experiments were performed on a confluent ECs monolayer. Cells were seeded at 150.000 cells/cm^2^ (bEnd5) or 100.000 cells/cm^2^ (MBMEC, HBMEC) and cultured for 3–7 days with replacement of medium once during that period to reduce handling stress. If seeded on inserts (ThinCert™, 1 μm pore diameter, 6–24 well plates, Greiner Bio-One, Lörrach, Germany), precoating with fibronectin (5 μg/cm^2^, 30 min, Sigma-Aldrich, St. Louis, MO, USA) was performed. If seeded on culture plates (Greiner Bio-One, 6-well), the wells were precoated with gelatine (0.1%, 30 min). For Western blotting and qPCR experiments treatment was started after establishment of a confluent ECs monolayer.

### Isolation of primary brain microvascular ECs

For isolation of MBMECs a previously described, modified protocol was used [25]. Briefly, after isoflurane anesthesia C57BL/6-WT-mice (female, 7-12w) were cervically dislocated and brains were harvested. After dissection of the cerebellum and olfactory bulb, removal of meninges was performed by rolling the brains on an autoclaved Whatman filter membrane (Schleicher & Schuell, Dassel, Germany). For each biological replicate, 5–7 brains were pooled and homogenized in buffer A [25] using a Dounce homogenizer (0.025 mm clearance, Wheaton, Millville, NJ, USA) and centrifuged at 400x g for 10 min at 4 °C.The supernatant was discarded and the pellet was digested with 0.75% collagenase II (Worthington, Lakewood, NJ, USA) in buffer A [(1,1:1 volume ratio; 1 h; shaking; 37 °C). After centrifugation (400x g, 5 min, RT) and aspiration of the supernatant, the pellet was resuspended in 25% BSA, centrifuged at 2000x g (30 min, 4 °C) in order to remove the myelin layer. After enzymatic digestion of the pellet with collagenase/dispase (1 mg/mL, Roche, Basel, Switzerland) and DNase I (1 μg/mL, Worthington) in buffer A (15 min, 37 °C) followed by centrifugation (400x g, 5 min, RT), MBMECs were resuspended in MCDB-131 complete medium [23] and seeded on 6-well plates pre-coated with collagen type 1 (100 μg/cm^2^, Corning, Tewksbury, MA, USA). Pure EC cultures were assured by puromycin treatment (5 μg/mL, MCDB-131 complete medium; 48 h) as ECs resist puromycin through their endogenous permeability-glycoprotein activity whereas other cell types are killed by the substrate. The puromycin-treated medium was replaced by standard medium and cells were used for the experiments afterwards. For isolation of HBMECs, unaffected cortex tissue from patients suffering from epilepsy (Additional file 3: Table S1) were obtained from the Department of Neurosurgery (university hospital, Frankfurt am Main) directly after operation. After cutting, samples were checked by an experienced neuropathologist (MM, PNH) and processed directly following the same protocol as described above, starting with homogenizing the samples in buffer A using a Dounce homogenizer. Afterwards, cells were deep-frozen (liquid nitrogen) and used for the experiments.

### Adhesion assay

Three biological replicates of bEnd5 cells were grown to build a monolayer as described above using a 24-well-plate (Greiner bio-one). After treatment with DTX (24 h, 5 ng/mL, ctrl., DMSO 1:1000) bEnd5 cells were washed 2x with PBS and 1000 MDA-MB-231-BR-GFP cells in MCDB 131 full medium were added to the insert and put in a humidified incubator for 70 min before being washed (3x with PBS) to remove the non-adherent cells and fluorescent signal was counted representing the remaining adherent GFP-expressing BR231 cells.

### Transendothelial electrical resistance (TEER)

MBMECs were isolated as described above and seeded (100.000 cells/cm^2^) on fibronectin-coated (5 μg/cm^2^, 30 min, Sigma-Aldrich) inserts (Greiner Bio-One, ThinCert™, 1 μm pore diameter, 24 well plates). After transfer to the cellZscope® device (Nano-Analytics, Münster, Germany), placed in a humidified incubator (37 °C, 5% CO2), TEER values were obtained from continuous impedance measurements as described previously [23]. After reaching a plateau in TEER levels (establishment of the endothelial-cell-monolayer) cells were treated with different concentrations of DTX (5 ng/mL; 500 ng/mL) for 48 h to 96 h. Statistical analyses were performed using Prism 6.0 (GraphPad Software, San Diego, CA, USA), paired t-test.

### Permeability assay

bEnd5 cells were cultured on 24-well inserts to build a monolayer as described above, before being treated with DTX (5 ng/mL) or DMSO-control (0.25%) for 72 h. Afterwards, for permeability assay, fluorescent tracers of different sizes (0.45 kD LY (Sigma-Aldrich), 3 kD TXR dextran (Thermo Fisher Scientific, Dreieich, Germany), 20 kD TMR dextran (Sigma-Aldrich), 70 kD FITC dextran (Sigma-Aldrich)) were used as described previously [23] at the following timepoints: 1 h, 2 h, 3 h. Briefly, the tracer mix was added to the upper chamber and at each time-point media aliquots from both chambers were collected. Samples were read in a fluorescence plate reader (Tecan, Männedorf, Switzerland) at the corresponding tracer excitation/emission. Permeability was calculated as follows: bottom chamber fluorescence normalized to the apical chamber fluorescence with the ratio for the control condition set to 100% [25]. Statistical analysis was done using Prism 6.0 (GraphPad software), unpaired t-test.

### RNA isolation and quality control for qPCR

bEnd5 cells were cultured and treated equivalently to the permeability assay (72 h; DTX (5 ng/mL); DMSO-control (0.25%)). Total RNAs were extracted using the RNeasy Mini kit (Qiagen, Hilden, Germany) according to the manufacturers protocol. RNA purity was monitored using NanoDrop® ND-1000 spectrophotometer (Thermo Fischer Scientific).

### Real-time quantitative PCR (qPCR)

1 μg of total RNA was subjected to reverse transcription using RevertAid First Strand cDNA Synthesis Kit (Thermo Fisher Scientific). Quantitative PCR (qPCR) was carried out using Fast SYBR Green Master Mix and the MyiQ single color real-time PCR detection system (BioRad, Hercules, CA, USA). Relative fold change (rfc) was calculated using the ∆∆Ct method. Gene expression was normalized to house-keeping gene G6PDX. Detailed information about targets and primers is presented in Additional file 4: Table S2. The figures show data obtained from at least three independent experiments. Statistical analyes were performed using GraphPad Prism version 6.0 (GraphPad Software). Quantitative data was assessed for significance by unpaired student’s t-test between the control- and the experimental conditions.

### Protein extraction and quantification

After having built a monolayer on 6-well plates as described above, bEnd5 cells were treated with DTX (5 ng/mL) for 24 h or 72 h. After washing twice with ice-cold PBS, 200 μL/well HES-Buffer (10 mM HEPES, 1 mM EDTA, 250 mM sucrose, pH 7.4 with protease- and phosphatase inhibitor cocktail (HALT) 10 μL/1 mL HES) was added, followed by scraping with a cell lifter and transferring to a tube (Eppendorf, Wesseling-Berzdorf, Germany) with consecutive sonication (3 × 3 s, low power) and centrifugation. Isolated cell lysate supernatant was either used directly or deep frozen (− 80 °C) for later use. Protein concentration was determined using Pierce™ BCA Protein Assay Kit (Thermo Fisher Scientific) according to manufacturer’s protocol, with measurement being performed using Tecan plate reader at A562 wavelength. For Western blotting 20 μg protein/sample was used.

### Western blotting

Samples were solubilized in urea sample buffer (1X SB; 3X SB = 8.5 M Urea, 7.5% w/v SDS, 0.25 M Tris-base pH 6.8, dissolved in Millipore water to 400 μL, add: 5 μL 0.5% w/v bromphenol blue, 95 μL TCEP; mix: 2:1 (20 μg Protein/SB)) for 1.5 h (shaking, 30 °C). After loading the samples on polyacrylamide gels (7–12.5%) according to the molecular weight of the protein of interest, electrophoresis was performed in one of the two ways (1: 20 min 80 V, 1–1.5 h 120 V, RT or 2: 2-3 h 80 V, RT). Afterwards proteins were blotted on a nitrocellulose membrane in one of two ways: (1: 1 h 100 V on ice, RT or 2: 20 h 36 V on ice, 4 °C). After blocking step (1 h, RT, 1x Roti®-block, Carl Roth GmbH, Karlsruhe, Germany), membranes were washed (2x PBS-T, 1x PBS, 10 min as described in detail peviously [25, 26]) and incubated with the respective primary antibody overnight at 4 °C, shaking (Table 2). After repeated washing and incubation with horseradish peroxidase-labeled secondary antibody (1 h, RT, shaking), imaging was performed using the Odyssey imaging device (LI-COR Biosciences, Lincoln, NE, USA), using a chemiluminescence system with a Luminol kit for protein band detection. For repetition of immunostaining on the same membrane, stripping (15 min, Restore™ PLUS Western blot stripping buffer, Thermo Scientific, Rockford, IL, USA), washing and blocking was consecutively performed. For quantification of protein bands, Image Studio Lite Vers. 5.2 (LI-COR) was used. Pixel density was measured for each band, background subtracted, normalized to loading control for each protein and recalculated to control DMSO set to 100% for better visualization. For statistical analysis, a two-tailed unpaired t-test was performed in GraphPad Prism version 6.0 (GraphPad Software).

**Table 2:** Antibodies for Western blot (WB), immunohistochemistry (IHC) and immunofluorescence (IF) analyses

Antibody	Company	Catalog #	Dilution (WB/ IHC/ IF)
α-tubulin	Sigma-Aldrich	T6199	1:1000/ X/ X
β-tubulin	Abcam	ab6046	X/ X/ 1:200
β-actin	Abcam	ab8227	1:1000/ X/ X
Claudin-5 (rb)	Thermo Fisher Scientific	34–1600	1:500/ 1:200/ 1:200
Claudin-5 (ms)	Invitrogen	35–2500	X/ X/ 1:200
Zonula-Occludens-1	Thermo Fisher Scientific	61–7300	1:500/ 1:500/ 1:200
Occludin	Thermo Fisher Scientific	71–1500	2 μg/mL/ 1:50/ X
Occludin	Invitrogen	OC-3F10	X/ X/ 1:200
VE-Cadherin	Santa Cruz	Sc-6458	1:500/ 1:200/ 1:200
Tie-2	R&D	AF762	0.1 μg/mL/ 1:200/ X
pTie-2	R&D	AF2720	0.5 μg/mL/ 2.5 μg/mL
ABCC4	Cell signaling	12857S	1:1000/ X/ X
wide-spectrum CK	Abcam	ab9377	X/ 1:20/ X
anti-mouse IgG	Dianova	115–035-146	X/ 1:500/ X
GFAP	Dako	ZO334	X/ X/ 1:5000
Iba1	Wako	019–19,741	X/ X/ 1:500
Ang2	Thermo Scientific	PA5–27297	1:5000/ 1:800/ X
CD31 (rt)	Dianova	DIA-310	X/ X/ 1:200
DAPI	invitrogen	D1306	X/ X/ 1:1000

*ms* mouse, *rb* rabbit, *rt* rat

### bEnd5 cell-pellet generation and staining

After having reached a sub-confluent cell monolayer (T-75 cell culture flask, Greiner Bio-One) as described above, bEnd5 cells were treated with DTX (5 ng/mL) for 24 h or 72 h. After washing with PBS, accutase (5 mL, 15 min, Sigma-Aldrich) was added until detachment of cells was observed. PBS was added and cells were harvested for centrifugation (400x g, 5 min). The supernatant was discarded and PFA was added (4%, 4 mL, 48 h). Afterwards the cell pellets were processed using standardized protocols for FFPE-tissue, cut into 3 μm thick slices and placed on a microscope slide (SuperFrost, Thermo Fisher Scientific), heated to 41 °C for 20 min and stored at 37 °C overnight in an incubator, followed by staining as described above using the automated IHC slide staining system Discovery XT (Roche/Ventana, Tucson, Arizona, USA) with the antibodies and dilutions depicted in Table 2. The stained tissue-slides were analyzed for differential staining intensity and gross morphological changes, using a light microscope (Olympus, Hamburg, Germany) with consecutive acquisition of representative images.

### Chamber-slides staining

Nunc™Lab-Tek™ II Chamber Slide™ System Permanox® (Thermo Fisher Scientific) was used for culturing of MBMEC monolayer as described above. After forming a monolayer, cells were treated with DTX (500 ng/mL) vs. control (DMSO 1:1000) for 24 h or 72 h. Afterwards, the slides were washed and stained as described previously [26] using the following antibodies: VE-Cadherin, Claudin-5, Occludin, ZO-1, CD31, β-tubulin (Table 2). Briefly, after washing steps in PBS, cells were fixed using methanol (100%, − 20 °C, 4 min) or PFA (4%, RT, 10 min), blocked (30 min, PBS containing 0.5% BSA, 0.1% Triton X-100 and the same buffer for primary and secondary antibodies) and incubated with the respective primary antibody for 1 h (RT) and secondary antibody for 1.5 h (RT), then counterstained using DAPI (1:1000, 5 min, RT). Representative images were taken using Nikon 80i microscope (Nikon, Düsseldorf, Germany).

### RNA preparation and quality control for RNA-sequencing

Cells (MBMEC, HBMEC) were isolated as described above (pooling of 6–7 mouse brains for each biological replicate (C57BL/6-WT, 7 weeks, female) for a total of 3 independent experiments for each condition (HBMEC: DTX-treatment *n* = 3, DMSO-control n = 3; MBMEC: DTX-treatment n = 3, DMSO-control n = 3)). After EC-isolation, cells were washed with PBS and trypsinized to let them detach. Then, MCDB 131 full medium was added followed by centrifugation (3 min, 400x g). For each biological replicate the cell pellet was resuspended in medium and seeded on to 4 inserts (12-well-inserts), precoated with fibronectin (5 μg/cm^2^, 30 min, Sigma-Aldrich) at 100.000 cells/cm^2^. The insert comprised 800 μL medium in the upper chamber and 1.5 mL medium in the lower chamber. DTX-treatment (HBMEC: 50 ng/mL; MBMEC: 500 ng/mL) was started after cells were grown to a monolayer (3 days); controls (DMSO, 1:1000) were treated similarly. After 24 h treatment, the medium was discarded, cells were washed two times with cold PBS and RNA was isolated inside a sterile hood following the manufacturer protocol using RNeasy Micro Kit (Qiagen), with the following modifications: RLT-buffer plus was used with DTT (40 μM) and samples were homogenized by repeated pipetting and vortexing (30s). The RNA concentration was determined using Qubit 3.0 Fluorometer (Thermo Fisher Scientific) with the manufacturer’s RNA-Kit according to the standard protocol. RNA-Quality was determined by Bioanalyzer using the according RNA Kit, to obtain specific RIN (RNA Integrity Number) values for each sample.

### RNA sequencing and differential gene expression analysis

Libraries were prepared with 500 ng of total RNA using the TruSeq mRNA Stranded Library Prep Kit (Illumina, San Diego, CA, USA) according to the manufacturer’s protocol. Briefly, mRNA pulldown was performed using an oligodT primer attached to the magnetic beads. To preserve strand information, the second strand synthesis was performed with the incorporation of dUTP which in turn made sure that following the PCR amplification, only the first stand was amplified. The libraries were quantified using the Qubit dsDNA HS assay kit (Thermo Fisher Scientific) and an Agilent 2100 Bioanalyzer (Agilent, Santa Clara, CA, USA). The pooled library was sequenced on an Illumina NextSeq500 using the manufacturer’s instructions. Demultiplexing of the sequenced libraries was done using bcl2fastq (v2.18.0.12). Mapping was performed using star aligner (v 2.5.2b) and the count matrix was produced using the featureCounts function from the subread package (v 1.5.2), using mouse annotation v GRCm38.87 and human annotation v GRCh38.87. Differential gene expression was performed with DESeq2 (v 1.14.1) using default parameters. Based on the obtained PCA plots (Additional file 1**:** Figure S1) outliers were identified with data being further analyzed after consecutive exclusion, leading to the final cleaned data-set (HBMEC: DTX-treatment *n* = 2, DMSO-control n = 2; MBMEC: DTX-treatment *n* = 3, DMSO-control n = 2), which was further processed for significance and equally regulated genes between mouse and human. Briefly, the experimental gene-sets were filtered for significance with consecutive data reduction for genes with log2fc leading into the same direction for mouse and human samples, resulting in the final gene-set.

### Murine brain metastatic model

Eight to twelve weeks old female Balb/c nude mice (Harlan Olac Ltd., Shaws Farm, Blackthorn, Bicaster, UK) were treated according to different treatment-schedules prior to TC injection. To establish BM, 500000 MDA-MB-231 BR TCs in 0.1 ml PBS were injected into the left ventricle under isoflurane/O_2_ anesthesia. DTX was injected intravenously according to the treatment schedule. DTX effect studies (samples for electron microscopy (EM), IgG staining) were performed similarly, however limiting the treatment-schedule to the “multi tax” (5 times) and “no tax” group without TC injection. DTX (Taxotere, 20 mg/mL, Sanofi-Aventis, Frankfurt, Germany) was reconstituted as indicated in the manufacturer’s protocol, followed by additional dilution (1:10) in NaCl 0.9% (final concentration: 2 mg/mL). Final solution was i.v. injected, 10 mg/kg bodyweight equaling approximately 100 μL/mouse (mean body weight/mouse = approximately 20 g). Control mice were treated equally, receiving 100 μL/mouse in 0.9% saline per injection. Four weeks after TC injection, mice were euthanized under CO_2_ asphyxiation and brains were harvested.

### Immunohistochemistry (IHC) and immunofluorescence (IF)

Brains were formalin-fixed (in 4% PFA for 48 h) and cut into 5–6 consecutive coronal slices and paraffin embedded. Respective tissue blocks were cut into 3 μm thick slices and placed on a microscope slide (SuperFrost, Thermo Fisher Scientific), heated to 41 °C for 20 min and stored at 37 °C overnight in an incubator. Hematoxylin and eosin staining was performed according to a standard protocol. For IHC staining standardized protocols for using the automated IHC slide staining system Discovery XT (Roche/Ventana) were used including the following antibodies (Table 2): anti-IgG (115–035-146; 1:500, Dianova, Hamburg, Germany), anti-wide spectrum Cytokeratin (CK) (ab9377, 1:20, Abcam). Afterwards slides were counterstained with hematoxylin and mounted. IF-staining was manually obtained by following the previously described protocol [27] for the respective primary antibodies: GFAP (ZO334, 1:5000, Dakocytomation, Glostrup, Denmark) and Iba1 (019–19,741, 1:500, Wako, Osaka, Japan). Stainings were evaluated for tumor foci count (HE, IHC for CK) following two different approaches to minimize subjective interpretation bias. First, HE-stained slides were evaluated for unequivocally distinguishable tumor foci using a light microscope. Secondly, immunohistochemically wide-spectrum CK stained slides were analyzed using a light microscope with Stereo Investigator (Version 4.34 software, MicroBrightField Inc., Williston, VT, USA), counting of TC metastasis, marked by CK-staining with subsequent normalization of the number of foci count related to the counted surface area. IF-stained slides were visually analyzed for gross morphological and intensity changes within tumor bearing areas. Representative images of tumor-bearing areas were taken using Nikon 80i microscope (Nikon, Düsseldorf, Germany).

### Electron microscopy

For electron microscopy, brains were fixed overnight using 2.5% glutaraldehyde buffered in cacodylate. The embedding procedure comprised fixation in 1% osmium tetroxide, dehydration in a graded ethanol series intermingled by an incubation step with uranyl acetate (between the 50 and 90% ethanol step) and finally rinsing in propylene oxide. The specimens were then embedded in epoxy resins that polymerized for 16 h at 60 °C. After embedding, first semi-thin sections (0.5 μm) were cut using an ultramicrotome (Leica Ultracut UCT, Deerfield, IL, USA) with a diamond knife. Sections were stained with Toluidine blue, placed on glass slides and examined by light microscopy to select appropriate areas for ultra-thin preparation. Ultrathin sections (50-70 nm) were cut again using an ultramicrotome. Sections were mounted on copper grids and contrasted with uranyl acetate for 2-3 h at 42 °C and lead citrate for 20 min at room temperature. These samples were imaged and digital pictures were taken with a FEI Tecnai G2 Spirit Biotwin TEM (Hillsboro, OR) at an operating voltage of 120 kV. Representative images being taken with an Eagle 4 K CCD bottom-mount camera.

### In vivo injections and magnetic resonance imaging (MRI)

DTX or 0.9% saline was injected as a bolus over 30s into the tail vein of 3 female and 7 male 4–6 months old NOD/SCID mice with an average bodyweight of 25.6 g. In the first experiment, 3 mice received 10 mg/kg DTX intravenously (i.v.) [28] (2 mg/mL dissolved in 0.9% saline) once a week for 4 weeks (4 injections in total), while 3 control mice received 0.1 mL 0.9% saline i.v. once a week for 4 weeks. In the second experiment, 2 mice received 10 mg/kg DTX intravenously (i.v.) (2 mg/mL dissolved in 0.9% saline) every second day for 6 days (days 0, 2, 4 and 6; 4 injections in total), while 2 control mice received 0.1 mL 0.9% saline i.v. every second day for 6 days. In both experiments, dynamic contrast enhanced MRI (DCE-MRI) was performed approximately 60 min after the last injection. As a positive control for permeabilization of the BBB, 3 female mice were given 200 mg K16ApoE dissolved in 100 mL 0.9% saline in the tail vein over 60s, and DCE-MRI was obtained 15 min after injection. K16ApoE is a peptide consisting of apolipoprotein E and 16 lysine residues and its ability to permeabilize the BBB has been described previously [29]. MRI was carried out using a 7 Tesla small-animal horizontal MR scanner (Bruker BioSpin GmbH, Ettlingen, Germany), using a 72 mm quadrature transmit coil and a 4-channel mouse brain array receive coil. The animals were placed in prone position and the body temperature was maintained at 37 °C. T_1_ and T_2_ weighted spin echo scans were acquired prior to DCE-MRI to provide anatomical references. The T_2_ weighted scans were acquired in coronal positioning (TR/TE: 4000/48 ms, field of view (FOV): 2.00 cm, matrix size: 256 × 256, slice thickness: 1.00 mm, 7 slices and number of averages (NEX): 4, total scan time 6 min 13 s). The T_1_ weighted scans were acquired with the same geometry as the T_2_ weighted scans (TR/TE 1000/9 ms, and NEX: 4, total scan time 3 min 20s). The DCE-MRI sequence consisted of 900 repetitions of the FLASH protocol with the same geometry as the T_1_ and T_2_ weighted sequences (TR/TE: 15 ms/2.1 ms, NEX: 1, FA: 17, temporal resolution: 1 s and total scan time 16 min 12 s). 0.5 mmol/kg Omniscan (GE Healthcare, Little Chalfont, UK) was injected as a bolus during 20s through the tail vein using an injection pump (Harvard Apparatus, Holliston, MA, USA) 15 s after starting the DCE-MRI sequence.

The DCE-MRI data was analyzed using the Extended Tofts model, implemented in nordicICE v2.3.14 (Nordic NeuroLab, Bergen, Norway), using local arterial input functions (AIFs), obtained from adjacent arteries. Maps of Area Under the Curve (AUC) were generated in two regions of interest (ROIs; areas 14mm^2^ (Fig. 3e) or 65mm^2^ in the mouse brain sections. Mean and standard deviation values were calculated for each ROI, and potentially statistically significant differences in AUC values between DTX- or K16ApoE-receiving animals and corresponding 0.9% saline receiving animals were determined using a two-sided Student’s T-test, with a significance level of 0.05.

### Statistical analysis

Statistical analyses were conducted using Prism 6.0 (GraphPad software) or JMP 14 (SAS, Cary, U.S.A.). For statistical analysis, a *p*-value < 0.05 was considered as significant and depicted in the graphs as followed: * *p* < 0.05; ** *p* < 0.01; *** *p* < 0.001. Information about experimental repeats and employed tests is indicated in the corresponding method section or figure legend.

## Results

### Taxane treated mBC patients show higher incidence of CNS metastases

**Fig. 1:**
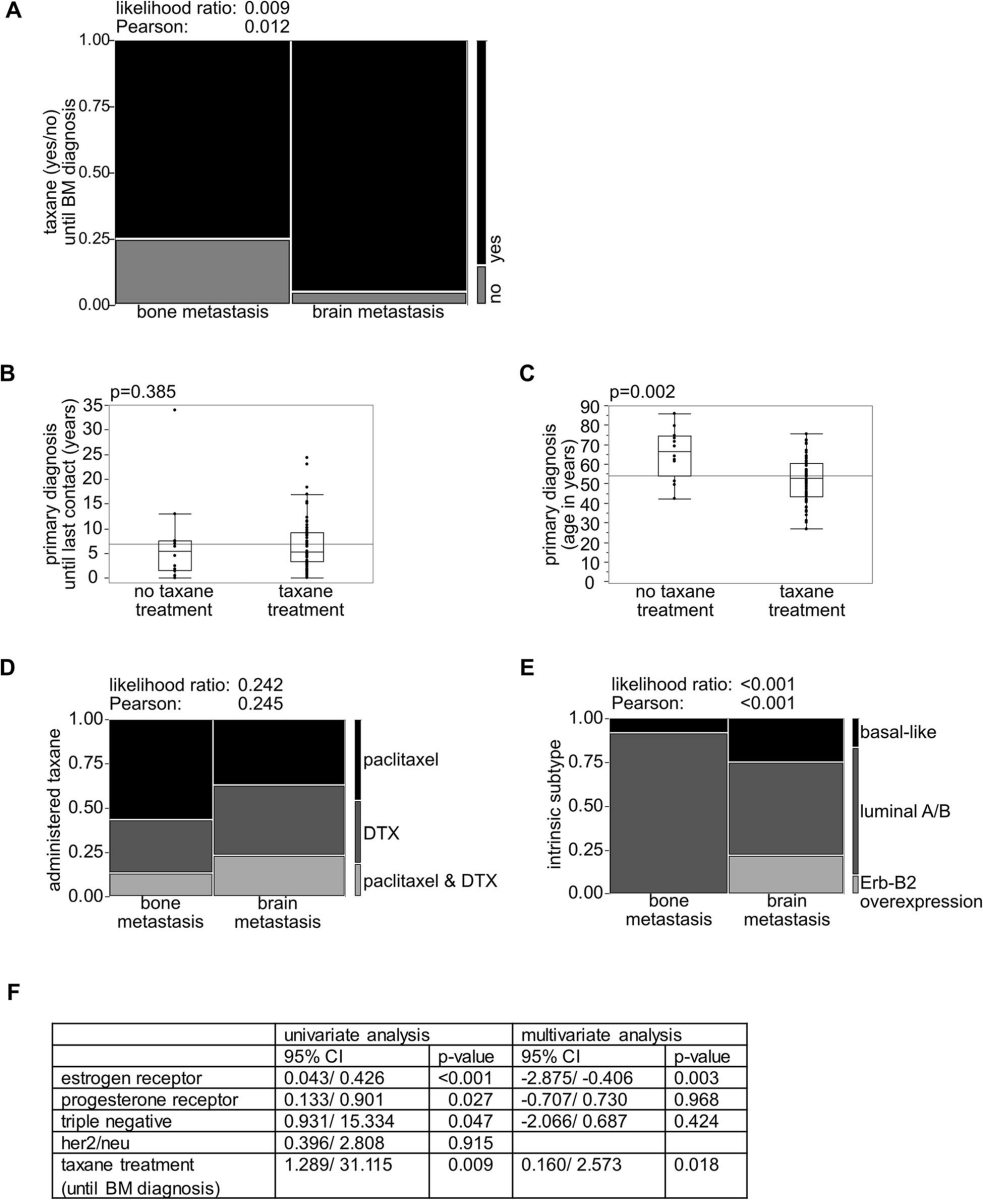
Incidence of CNS-involvement is increased in taxane-treated mBC patients. **a** Contingency analysis with likelihood-ratio and Pearson test of taxane-treatment (yes/no) for BM- vs. nBM-cohort. **b**, **c** Non-parametric multiple comparisons for each pair using Wilcoxon-method: **b** Follow up “primary diagnosis” (N (notaxane) = 12, N (taxane) = 68); **c** Patient age primary diagnosis (N (notaxane) = 12, N (taxane) = 68). **d**, **e** Contingency analysis with likelihood-ratio and Pearson test of (**d**) administered taxane (N (nBM) = 30, N (BM) = 38, N varies from 40 as the non-taxane-treated subcohort was excluded for the analysis) and (**e**) intrinsic subtypes (N (nBM) = 40, N (BM) = 40) for BM- vs. nBM-cohort. **f** For univariate analysis, effect likelihood ratio and odds ratio test was used; for multivariate analysis nominal logistic fit for the endpoint BM-development using effect likelihood ratio test was applied. Statistical analysis was performed using JMP 14.0.0 software (SAS)

The conflicting data about whether or not taxane treatment leads to an increased rate of BM prompted us to investigate our own patient cohort (*n* = 80). The patient characteristics and tumor biological parameters are summarized in Table 1. Patients were reviewed retrospectively according to a case-control approach: BM (BM, exp., *n* = 40) vs. no BM, but bone metastases (BoM, ctrl., n = 40), to test for the possibility that taxane-treatment (DTX or paclitaxel) may be associated with an increased development of BM. In our monocentric, BC patient cohort, patients who progressed to brain metastatic disease were found to having significantly more often received taxane-treatment in the course of their disease than the nBM- subcohort (Fig. 1a). Taxane-treated vs. non-taxane-treated (*n* = 68 vs. *n* = 12) patients showed comparable duration of follow-up since primary diagnosis (Fig. 1b). Also, there was no difference regarding the start of taxane treatment or follow-up related to the taxane treatment (Additional file 2: Figure S2A), but taxane-treated patients were significantly younger at their BC-diagnosis (Fig. 1c). BM and BoM patients were relatively similar concerning the administered taxanes, DTX/paclitaxel (Fig. 1d). BM and BoM patients did not differ in survival (Additional file 2: Figure S2B). Median interval between BC diagnosis to BM development was 4.879 years (Additional file 2: Figure S2C). However, the cohorts significantly differed concerning the BC intrinsic subtypes (Fig. 1e). BM patients were, in univariate analysis, significantly more often ER negative, PR negative and triple negative, but did not differ with regard to HER2/neu (Fig. 1f). Notably, ER being negative and taxane treatment remain the only significant risk factors for BM formation in the consecutive multivariate analysis (*p* = 0.003; *p* = 0.018) (Fig. 1f).

### DTX treatment increases CNS metastasis formation in a murine intracardiac TC injection model

**Fig. 2:**
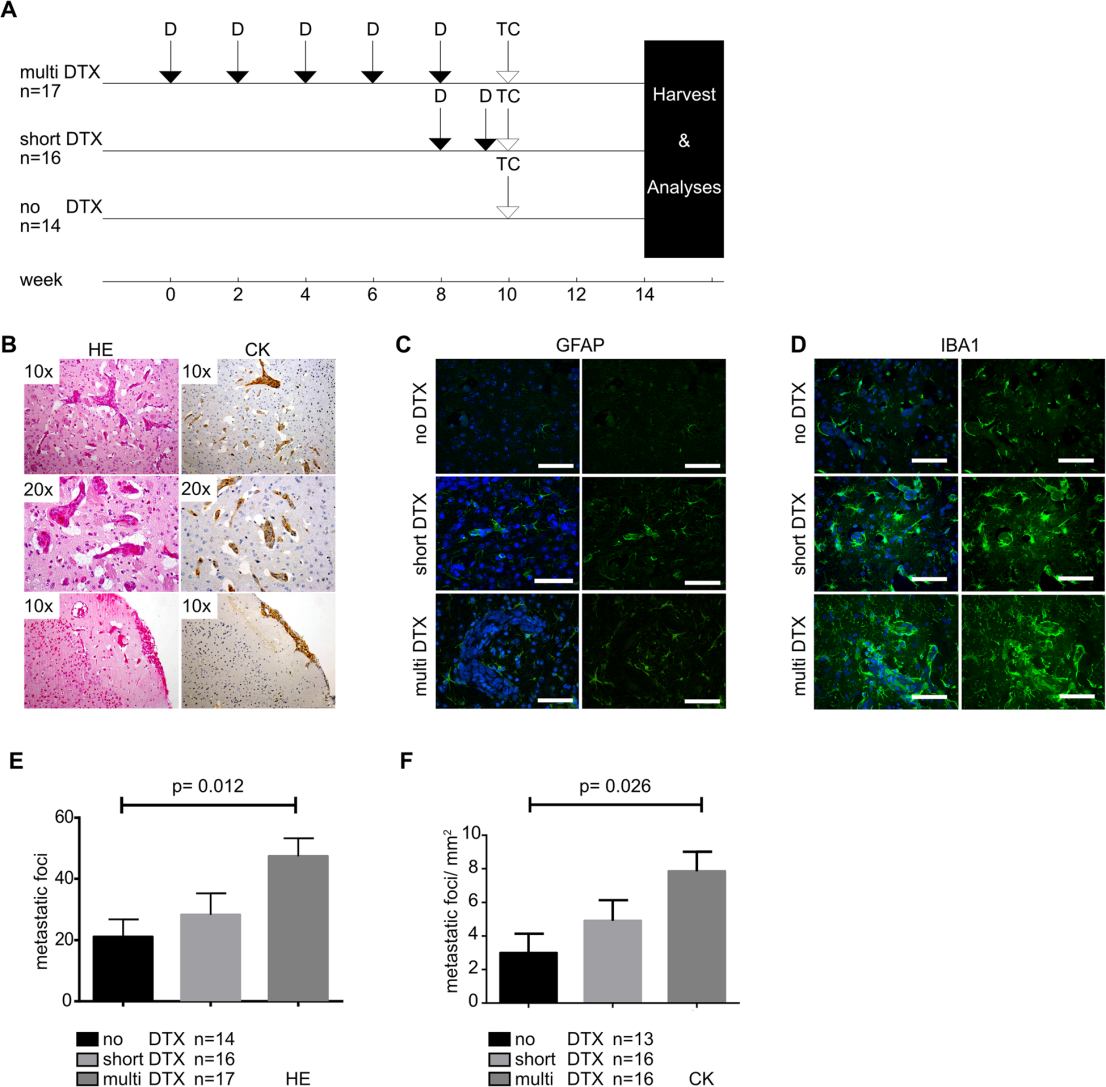
DTX-pretreatment in balb/c nude mice increases CNS metastatic load after intracardiac TCs injection. **a** Experimental setting of the animal model. **b** Representative HE and immunohistochemical (wide-spectrum cytokeratin) stainings of established tumor foci (original magnification 10x or 20x). **c**, **d** Representative IF-stainings of microenvironmental changes surrounding established tumor foci of different sizes and treatment groups: staining for (**c**) astrocytes (GFAP), **d** microglia (Iba1) (images taken with the Eclipse 80i fluorescent microscope; scale bar, 50 μm). **e**, **f** One way ANOVA with Kruskal-Wallis test for CNS tumor foci count performed either in (**e**) HE- or (**f**) wide-spectrum cytokeratin IHC-staining. Statistical analysis was done using GraphPad Prism software

To further study DTX effects on BM formation, we used a murine model in which mice were pretreated with DTX (10 mg/kg body weight) prior to intracardiac (left ventricle) injection of MDA-MB-231 BC cells, according to three different treatment regimens: multi DTX (5 times), short DTX (2 times), no DTX (Fig. 2a); followed by neuropathological assessment (Fig. 2b-e). Microscopic assessment of the murine brains confirmed a mainly perivascular infiltration pattern of the TCs, whereas a spread to the cerebrospinal fluid, or superficial metastases was rarely observed (Fig. 2b). Immunofluorescence (IF) analysis of astrocytes (GFAP) and microglia (Iba1) showed increased activation of both cell types surrounding tumor foci, without associated DTX-specific changes, corroborating a well working BM-model [30] and nicely mimicking BM distribution usually observed in patients [31] (Fig. 2b-d). Differences in BM foci were observed between the groups, with a significantly higher amount of BM in the “multi DTX” group as compared to “no DTX” (*p* = 0.012 (for HE); *p* = 0.026 (for CK)), and a strong trend as compared to “short DTX” (Fig. 2e, f).

### DTX treatment does not impair BBB permeability in-vivo

**Fig. 3:**
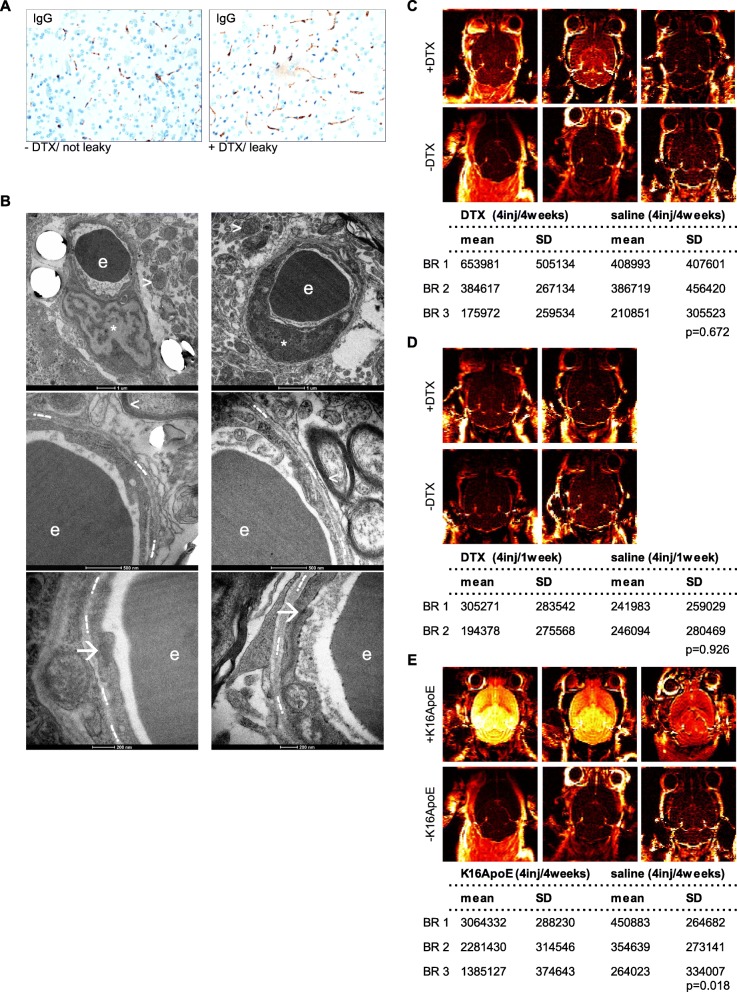
Permeability analyses of DTX-treated mice in-vivo do not show signs of increased leakage. **a** Exemplary IHC-stainings of murine IgG using brain sections of DTX-treated vs. control mice (original magnification 20x). **b** Representative images of ultrastructural NVU-imaging: ECs (indicated by *); TJs (indicated by ➔); perivascular axons (indicated by <=; mitochondria (indicated by >); basement membrane (indicated by .--.); erythrocyte (indicated by e). Images taken using Tecnai Spirit BioTWIN FEI EM at 120 kV, with 4 K CCD camera. **c**-**e** DCE-MR imaging heatmaps of treated vs. untreated mice for in-vivo permeability analysis. Statistical analysis was conducted using student’s t-test, subset analysis for three different groups as depicted in the methods section

As DTX treatment enhances BM-formation and brain blood vessel homeostasis and integrity is mainly provided by the BBB [32], we next investigated BBB properties and potential DTX-induced BBB alteration in mice in-vivo by means of IHC, EM and DCE-MRI. First, light microscopic immunohistochemical IgG-staining of DTX-treated mouse brain showed no change of permeability as compared to controls (Fig. 3a). Along this line, ultrastructural analyses of DTX-treated mice revealed no relevant changes of the NVU such as unequivocal disruption of tight-junctions or relevant morphological alterations of endothelial cells (ECs) or respective organelles (Fig. 3b).

In our DCE-MRI analysis, we first compared animals receiving either 4 i.v. injections of DTX over 4 weeks, with control animals receiving 4 i.v. injections of PBS over 4 weeks. No statistical differences in AUC could be found (*p* = 0.672; Fig. 3c). We then treated animals with 4 i.v. injections over 6 days, either with DTX or PBS. Also here, no differences in AUC could be found (*p* = 0.926, Fig. 3d), whereas positive control animals (one injection of K16ApoE) showed a prominent permeabilization of the BBB (*p* = 0.018, Fig. 3e). Transient local permeability changes may be rather difficult to detect in-vivo, so we next performed in-vitro experiments.

### DTX-treated ECs do not lead to more adherence of TCs

**Fig. 4:**
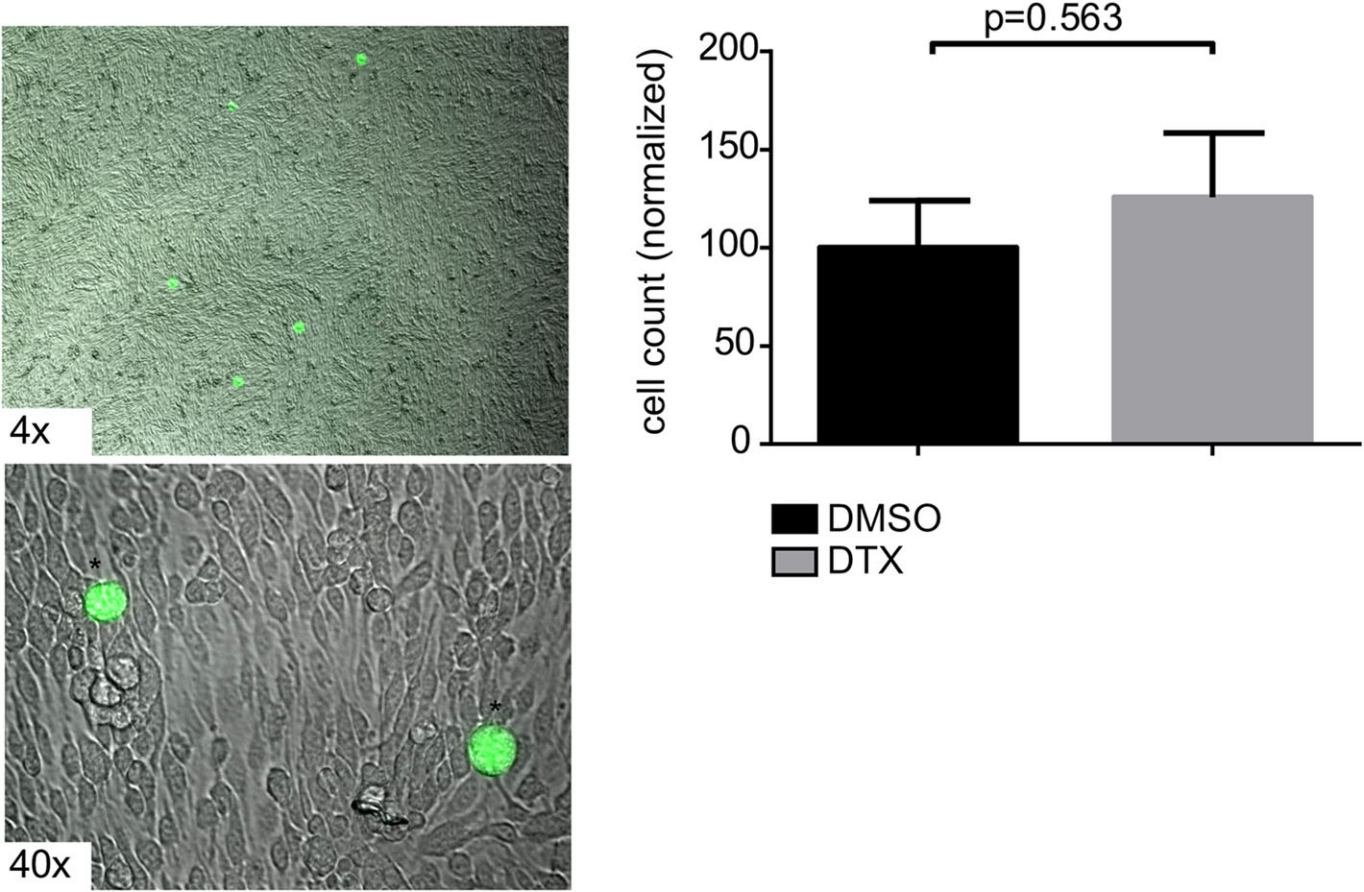
TCs do not show increased adhesion on EC monolayer upon DTX treatment**.** Representative images of the adhesion assay showing GFP-labeled (*) MDA-MB-231-BR-GFP-TCs on top of ECs monolayer. Phase-contrast microscope with IF-imaging, original magnification 4x, 40x. Unpaired t-test of treated (*N* = 3) vs. untreated (N = 3) bEnd5 cells monolayer, with TCs plated on top. Statistical analysis was done using GraphPad Prism software

Regarding the metastatic cascade, adhesion and subsequent transmigration take place during BM-formation [33]. Therefore, we checked if DTX-pretreated ECs may lead to an increased adhesion of MDA-MB-231 TCs (GFP-labeled) plated above the ECs, however no significantly enhanced TC adhesion could be observed in the ECs that have been treated with DTX (Fig. 4).

### In-vitro BBB-permeability is increased upon DTX-treatment with concentration-dependent kinetics

**Fig. 5:**
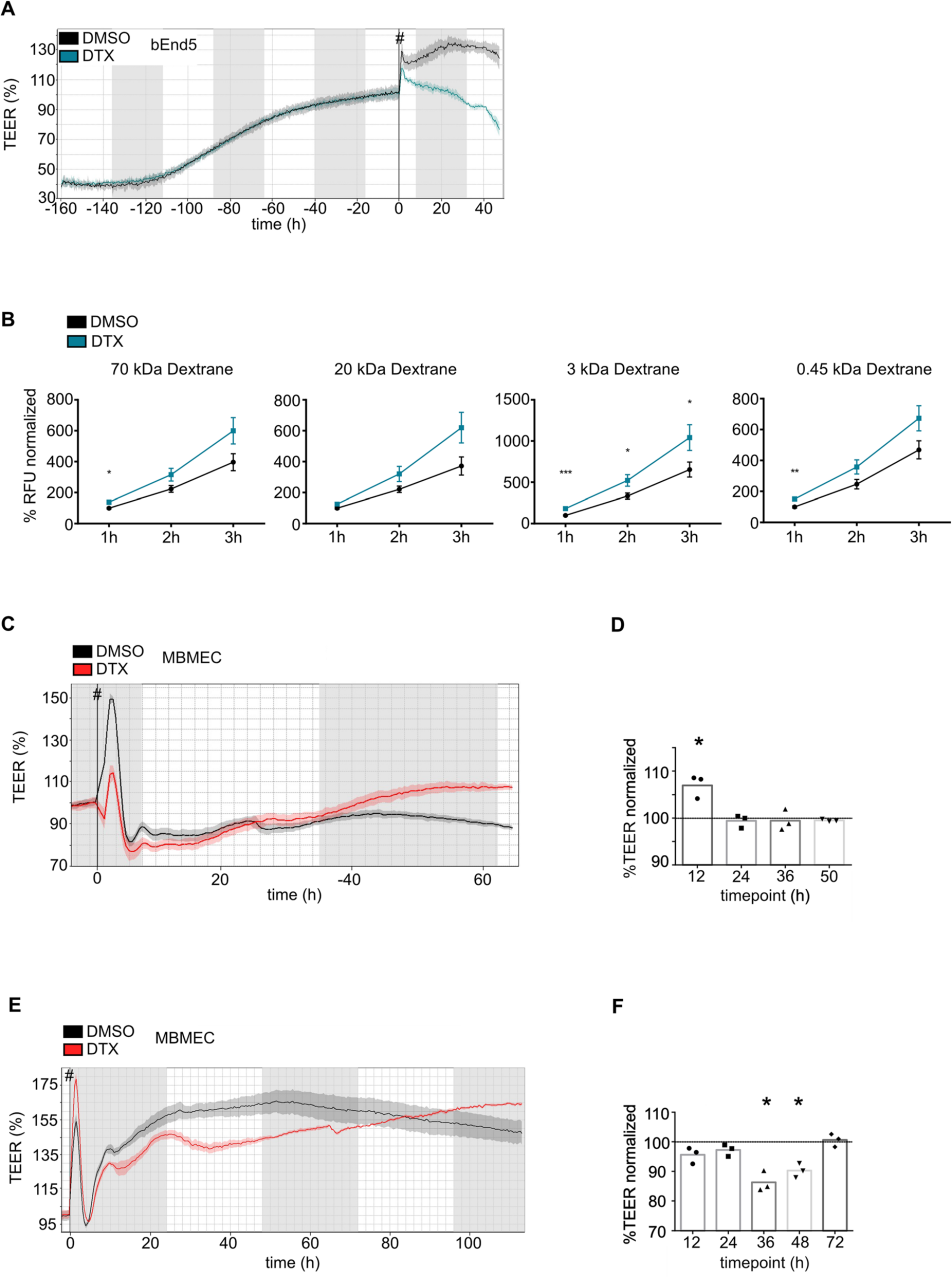
DTX-treatment increases BBB-permeability in-vitro in a concentration-dependent manner**. a** Representative image of bEnd5 cells monolayer in TEER-measurement. **b** Permeability assay of treated (N = 3) vs. untreated (N = 3) bEnd5 cell monolayer, using different sized tracers (kDa 0.45; 3; 20; 70). Statistical analysis: unpaired t-test using GraphPad Prism Software. **c** Illustration of TEER-curve progression using primary MBMECs with DTX-treatment ((**c**) 5 ng/mL; **e** 500 ng/mL) vs. control and subsequent statistical analysis with GraphPad Prism software, using paired t-test (**d**, **f**). start of treatment, #

An in-vitro BBB-model was applied to address a potential transient DTX impact on the BBB. BBB-permeability was assessed with TEER-measurements, a sensitive method to study EC-monolayer integrity and permeability [34], with loss of resistance paralleling increased permeability (Fig. 5). TEER-measurements showed a continuous increase of resistance until reaching the plateau phase, where the treatment was started, which resulted in a steady decrease of resistance in DTX-treated bEnd5-cells within our observation frame (Fig. 5a). Also, permeability to different sized fluorescent-labeled agents (kDa: 70; 20; 3; 0.45) was increased in DTX-treated bEnd5 cells. Two tracer sizes (kDa: 70; 0.45) showed significant increase in permeability for one and a strong trend for the other time points. The 3 kDa-tracer was significantly altered for all time points and the 20 kDa tracer was not significant but showed a strong trend for all time points (Fig. 5b). Those findings led us to verify the results using primary MBMECs and we found DTX-treatment leading to concentration-dependent permeability changes; showing a minimal, non-significant trend at the dose of 5 ng/mL-DTX (Fig. 5c, d), and a stronger TEER-decrease at 500 ng/mL-DTX, with leakage being significant around 36–48 h (36 h *p* = 0.021; 48 h *p* = 0.02) after the treatment, and being again absent with a trend to even tightening the BBB after 72 h (Fig. 5e, f).

### Unaltered tight junction protein expression and a delayed increase of VE-cadherin protein expression, upon DTX treatment

**Fig. 6:**
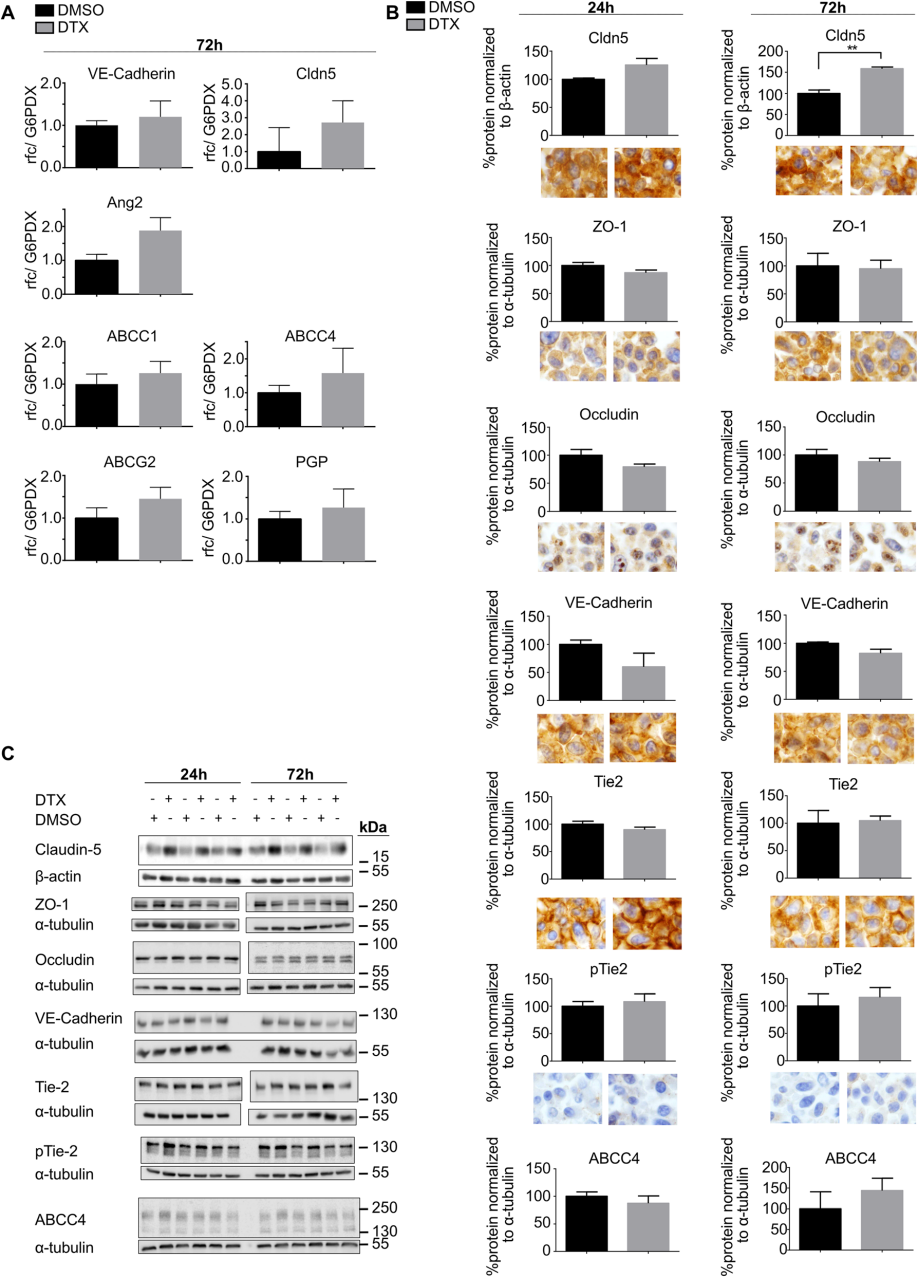
qPCR, WB and ICC target analyses of DTX-treated bEnd5-cells. **a** Differential mRNA expression analysis by quantitative polymerase chain reaction (qPCR) of three independent experiments, treated (N = 3) vs. ctrl (N = 3) ECs, using unpaired student’s t-test, GraphPad Prism software. **b** Analysis of WB data (N = 3 treated vs. N = 3 ctrl) using unpaired student’s t-test. Exemplary images of respective bEnd5 cell-pellet IHC-stainings (original magnification 40x). **c** Respective immunoblots used for statistical analyes of WB data, each pair of −/+ represents a biological replicate

We next analyzed possible molecular components of DTX-induced increase in permeability in-vitro, by qPCR, WB and immunostainings of ECs (Fig. 6). Selected candidates were first screened by qPCR. A trend towards increased expression upon DTX treatment was observed for the junctional molecules VE-Cadherin and Claudin-5, as well as for a variety of pumps, known to play important roles in ECs [24] (Fig. 6a). The strongest trend of increased expression was shown for Angiopoetin-2 although not reaching the threshold level of significance (*p* = 0.133; Fig. 6a). Next, appropriate candidates were analyzed by WB. With regard to 24 h DTX-treatment, no significant change of protein levels could be observed for any proteins including those involved in tight-junctions (ZO-1, Occludin) (Fig. 6b, c). The proteins known to be able to alter BBB-permeability, related to the trend of increased Ang2-level, with the respective axis of Tie2 and pTie2, were not altered [35] (Fig. 6b, c). Also the MDR-pump ABCC4, as important BBB efflux transporter [24, 36], was not affected (Fig. 6b, c). 72 h-treatment paralleled above effects, with the exception of Claudin-5 being significantly upregulated in the DTX group (Fig. 6b**, c**). Further, microscopic analysis of IHC-stained ECs cell-pellets did not show distinct DTX-treatment related protein alterations, supporting the WB data (Fig. 6b).

### Altered endothelial ß-tubulin distribution and nuclear morphology but not BBB associated molecules upon DTX treatment

**Fig. 7:**
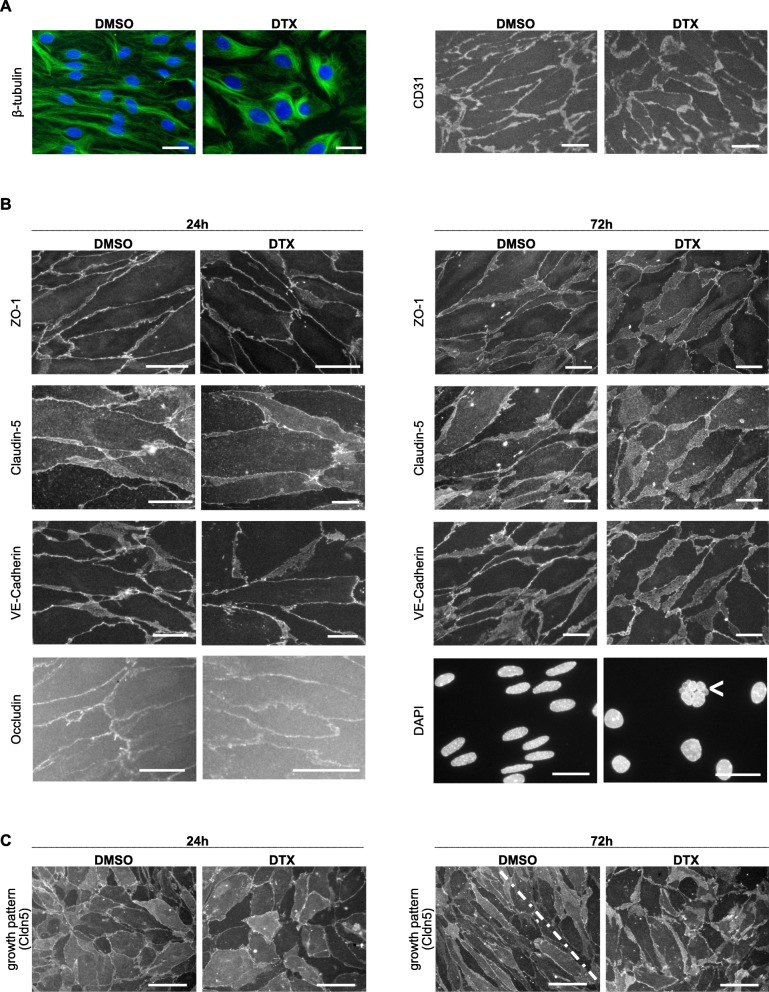
IF analyses of junctional BBB signature-proteins in MBMEC-monolayer. Representative IF-stainings of signature BBB-proteins. **a** 24 h treatment; **b**, **c** 24 h and 72 h treatment as depicted, (**a**, **b**) scale bar, 20 μm; karyorrhexis, <; **c** scale bar, 50 μm

As junctional protein-levels were not changed, we investigated if their intra−/ intercellular distribution, organization or morphology may be altered using IF-stainings of primary MBMEC monolayers with EC-origin being assured by comprehensive CD31 marker expression (Fig. 7a). DTX-treatment resulted in disturbed, coarse tubulin-morphology (Fig. 7a). A uniform monolayer was established in both groups associated with a global expression of junctional proteins (Fig. 7). IF microscopic analysis revealed no relevant changes of morphology, distribution or organization of the analyzed proteins, although DTX treatment impaired elongation of EC nuclei, induced occasional karyorrhexis and led to reduced EC density (Fig. 7b). Treatment was started when the monolayer mainly presented in an unorganized growth pattern and interestingly, after 72 h, the control group displayed already large areas of typical spindle shaped morphology of mature BBB forming ECs, whereas the DTX-group comprised mainly an disorganized coarse pattern lacking smooth cell-cell borders (Fig. 7c).

### Human and murine DTX-treated ECs show a common candidate gene set potentially involved in EC alteration

For deeper analysis of the underlying mechanism on the molecular level, RNA-sequencing was performed using primary cultured brain ECs from mouse (MBMEC) and human (HBMEC) after 24 h of DTX treatment in-vitro. First, mRNA-sequencing-data were processed for differential expression (DeSeq2), then overlapping significantly and equivalently regulated genes from mouse and human were analyzed (Table 3). Similar regulation was found for different members of the tubulin family (β 2B, β 2A, α 4A, α 1A) which are involved in forming the microtubule-structures, being upregulated and only one other protein-coding gene, namely TSPAN2, which codes for Tetraspanin-2, which was downregulated (log2foldchange: ms = − 0.475; hu = − 1.513) (Table 3).

**Table 3:** RNA sequencing data showing significant changes in expression mouse (MBMEC) and human (HBMEC) endothelial cells

	mouse		human	
	log2fc	p-value	log2fc	*p*-value
Tubb2b	1.315	< 0.001	1.725	< 0.001
Tubb2a	1.212	< 0.001	1.523	< 0.001
Tuba4a	1.472	< 0.001	1.231	< 0.001
Tuba1a	1.231	< 0.001	1.364	< 0.001
Tspan2	−0.475	0.028	−1.513	0.007

Differential expression analysis of mRNA levels of mouse (DTX, n = 3; ctrl, n = 2; DTX, 500 ng/mL) and human (DTX, n = 2; ctrl, n = 2; DTX, 50 ng/mL) primary cultured ECs after 24 h of DTX-treatment. Analysis was done using DESeq2 package, based on PCA plot. Outliers were excluded resulting in the above stated n

## Discussion

The brain displays the most dramatic site for cancer metastasis [37] with limited available therapeutic approaches [37, 38]. Therefore it is of importance to identify risk factors, leading to BM formation [37]. Though controversial data exist concerning a possible increase of CNS-involvement in BC-patients treated with a chemotherapeutic agent of the taxane family, the question whether or not taxane treatment may alter BBB-properties, facilitating TC transmigration into the brain and thus establishing of BM, was never experimentally addressed [20–22]. Our results demonstrate a positive association between taxane treatment and BM formation with a significantly increased BM rate in both BC patients and a BC mouse model, suggesting a direct effect of taxanes on BBB function.

Previous studies proposed the CNS as sanctuary site for TCs [13, 21], showing that adjuvant chemotherapy may lead to a higher frequency of BM [21, 39]. Also a transient chemotherapy induced BBB-alteration, which might facilitate TC transmigration across the BBB into the brain parenchyma is discussed [4].

Controversial results have been reported about the question if chemotherapeutic agents from the taxane family lead to an increase of CNS-relapse in BC-patients: (i) high rate of CNS-relapse (17.9% of initial treatment-responders) was seen by Freilich et al. [21], who investigated 152 patients with different doses and schemes of paclitaxel therapy being applied. However, no control group was analyzed in this study; (ii) high frequency of CNS-involvement (30.4%) was shown by Crivellari et al. [22], investigating 92 patients being treated with Epirubicine and DTX, yet also this study missed an adequate control group and (iii) no increased BM-frequency (no taxane-treatment: 4%; taxane-treatment: 3.7%) was shown in the study of Pestalozzi et al. [20], investigating 2887 npBC patients prospectively, however results were limited by study design as for CNS-relapse analysis only the 403 patients who died within the follow up of 5 years were investigated, resulting in 110 patients with BM; patients suffering from non-symptomatic BM were not included. As literature findings describe CNS involvement in breast cancer patients as being highly heterogenous, ranging from 3.9–20% (or even up to 30.4%, Crivellari et. al) [22, 40], a valid power analysis of our patient cohort was not achievable. In our small, monocentric patient cohort, taxane treatment was significantly positively associated with BM formation as compared to a bone metastatic control group (Fig. 1a). Patients receiving taxanes were significantly younger (Fig. 1c) and BM patients were significantly more often TN, PR- or ER negative (Fig. 1f), all factors known to be associated with increased BM development [41, 42]. Also, the cohorts differed with regard to the BC intrinsic subtypes (Fig. 1e), with the BM-cohort incorporating significantly more often basal-like- and Erb-B2 overexpressing subtypes which are known to show a high rate of BM [43, 44]. We did not include the Ki-67-proliferative index, because no generally accepted, comprehensive recommendation for standardization is available yet, which let us to combine the intrinsic subtypes Luminal A and -B into one subtype [15, 44]. We performed a multivariate analysis with taxane treatment still being significantly associated with an increased risk of BM formation while the other factors, except ER being negative, were no longer significant (Fig. 1f). Nevertheless, our cohort suffers from major limitations such as its retrospective design, incomplete data for some patients, a rather small sample size, significant differences in tumor biology and intrinsic subtypes, non-significant differences in survival, as well as the administration of a broad spectrum of medication. Taken together, the epidemiologic finding of DTX-effect (Fig. 1) is critical and needs to be discussed but should not be overstated. The question if taxane treatment is a relevant factor for increased BM development and may confer single TCs an increased probability to cross the BBB cannot be answered by analyzing patient data only. Therefore, we used a previously described murine BM model [45]. To study DTX effects on blood-vessels, without being biased of sanctuary TC growth [13, 22], mice were pretreated with DTX prior to TC injection, therefore interactions between DTX and TCs can be ruled out in our setting (Fig. 2a). We used MDA-MB-231-BR-GFP cells as they have a tropism to metastasize to the brain [45]. Our results demonstrated an increased amount of BM foci paralleling increasing DTX levels with a mainly perivascular infiltration pattern (Fig. 2e, f), nicely mimicking brain metastatic cooptive growth pattern [33, 37] (Fig. 2b).

Cells can pass the BBB either via paracellular or transcellular routes [33]. The former requires that the cells pass through intercellular junctions, which is facilitated when permeability is increased [33]. However, we could not observe relevant alterations of the BBB permeability upon DTX treatment in-vivo (Fig. 3). Prior to transmigration, TCs need to attach to the ECs in a selective manner or via a mechanical arrest, similar to a thrombus plugging the blood vessel in ischaemic stroke [7, 46, 47]. Therefore, we hypothesized that DTX may lead to increased adhesion in-vitro, but also here, no differences were observed (Fig. 4). Of note, not a single human tumor cell showed plane adhesion nor migration upon or through the murine EC monolayer pointing to interspecies differences [48]. Consequently, our murine metastatic model might not be able to reveal this specific adherence step of the metastatic cascade which may hereby be ruled-out as being crucial for the observed increased BM-frequency. In our model, intravascular tumor cell arrest most probably occurs mechanically by plugging the vessel and subsequent transmigration [7]. It is known that TC transmigration damages ECs, leaving apoptotic cells and a debilitated barrier behind, a suitable entry point for close-by TCs [49, 50]. Furthermore, DTX impacts endothelial proliferation-status, reduces wound-healing capacities [51, 52] and sensitizes ECs to hypoxic damage [53], all factors potentially facilitating TC transmigration and thereby increasing BM foci.

Considering the possibility of insufficient sensitivity of hitherto in-vivo BBB-permeability analysis, we used an in-vitro BBB model with continuous TEER-measurement, known to be a sensitive and reliable method to analyze EC-monolayer tightness and integrity [24, 34] (Fig. 5). As TEER only measures permeability to ions [25, 54], we also checked permeability with regard to the tracers of different sizes, which was increased either significantly or with a strong trend after DTX treatment. First, to keep the animal number as low as possible, we used immortalized murine ECs (bEnd5), being a suitable cell-line for BBB-analyses [24]. Further, we corroborated those findings using primary MBMECs for TEER-measurements, as they display best in-vitro*/*in-vivo comparability [24, 36], revealing BBB-impairment positively correlating with the used DTX concentration in time and extent, to finally being hypercompensated, leading to an increased tightening of the BBB. We further analyzed a subset of previously described BBB signature markers [24, 33, 36, 55] to elaborate possible molecular components associated with DTX-associated permeability increase in-vitro, including the Ang/Tie2-axis which is known to have an impact on vessel integrity, with Ang2 contributing to vessel destabilization [35] (Fig. 6**)**. Nevertheless, the weak trend of increased Ang2 mRNA-levels could not be corroborated at protein level. There were no changes which would indicate an unequivocal BBB leakage, however the significant increase of Claudin-5 after 72 h treatment could be interpreted as being part of a compensatory tightening effect (Fig. 6b, c). We also investigated a potential impairment of junctional protein distribution, arrangement and morphology (Fig. 7). It has been shown for epithelial cells that besides actin [56], microtubules are essential for TJ homeostasis and restoration [57]. Prior studies reported an attenuated disassembly of epithelial and endothelial junctional proteins upon taxane-induced microtubule stabilization [58–60]. Further, disruption of microtubules significantly reduced barrier functions in TEER-assays, highlighting the importance of interaction between microtubules in junctional preservation [57]. Our immunostaining-based morphologic analysis proved tubulin-affection, whereas junctional proteins did not show relevant changes. Interestingly, DTX treatment inhibited induction of the organized ECs growth pattern being in line with previous studies showing that DTX treatment impairs EC migration [52] and attenuates junctional disassembly [58]. This may also explain the attenuated barrier compensational capacities, especially after EC-damage.

Based on the stated functional findings but scant identification of molecular targets, we went for mRNA-sequencing. The unexpected finding that most of the significantly regulated genes did not match between mouse and human ECs, let us to interpret the matching genes as being the most important, obtaining 5 significantly regulated genes (Table 3). Various members of tubulin families were upregulated upon DTX treatment, which served well as proof of principle of achieved DTX effect [61, 62]. The only otherwise similarly downregulated gene was Tspan2 coding for the protein Tetraspanin-2. Tetraspanin-2 has indeed reported as being involved in cancer metastasis and tumor-related angiogenesis [63, 64]. In tumor-conditioned ECs, epigenetic silencing of Tspan2 was identified as a driver of angiogenesis corroborated by the direct angiostatic effect caused by DNA methyltransferase and histone deacetylase inhibitors-treatment [64]. Additionally, knockdown of Tspan2 increases ROS production [63], similarly to DTX [65]. Increased ROS production might therefore constitute a potential mechanistic link between Tspan2 and DTX treatment, since it affects the BBB permeability among others through TJ protein modulation [66–68]. Occludin, a crucial tight junction molecule of the BBB which has been implicated in BBB dysfunction in hypoxia and ischemic stroke, also belongs to the tetraspanin family [32, 36]. The role of tetraspanin-2, obtained from our sequencing analysis could potentially be a novel candidate regulating the BBB function [32, 36] . These aspects are in line with our stated hypothesis: tumor cell clots leading to thrombotic occlusion of blood vessels with consecutive hypoxia of endothelial cells, that are sensitized to BBB-damage through DTX treatment thus showing an increased BBB-impairment, may facilitate tumor cell transmigration to the CNS. Additionally, DTX-induced attenuated TJ dynamics prolong junctional-recovery [57–60], leading to a longer time frame of barrier dysfunction may also facilitate tumor cell transmigration. Finally, the cascade is potentiated by the pro-angiogenic state of the ECs. The stated, mechanistic hypothesis was not further analyzed, therefore needing further investigation.

## Conclusion

In conclusion, DTX treatment seems to increase BM rate in human and mice, hypothesizing a direct effect of taxanes on BBB properties. This effect seems to be rather transient (TEER), followed by a hypercompensatory (TEER, WB data Claudin-5) state, however with no morphologically detectable long-term changes (EM, IgG-IHC). We could not identify a definite mechanism how DTX treatment impairs BBB properties, however particularly molecules of the tubulin-family and tetraspanin-2 seem to be involved. A tight neuroradiologic follow-up for mBC-patients receiving taxane is proposed and further investigation is needed.

## Supplementary information

**Additional file 1: Figure S1**. Principal component analysis (PCA) of RNA-Sequencing data.

**Additional file 2: Figure S2**. Start/end of taxane-treatment, follow-up and survival did not differ between BM/ BoM patients. (A) Non-parametric multiple comparisons for each pair using Wilcoxon-method: Years: primary diagnosis until first taxane (N (nBM) = 23, N (BM) = 27); Follow up “first taxane” (N (nBM) = 23, N (BM) = 28); Follow up “last taxane” (N (nBM) = 29, N (BM) = 34). (B) Kaplan-Meier survival curves of BM vs. BoM patients. Curves were compared by log-rank and Wilcoxon tests. (C) Years: primary diagnosis until BM (N (BM) = 38).

**Additional file 3: Table S1.** HBMEC Patient characteristics.

**Additional file 4: Table S2.** Primers used for qPCR analysis.

## Data Availability

The datasets supporting the conclusion of this article are included within the article and its additional files. Please contact the author for additional reasonable data requests.

## Abbreviations

BBB: Blood-brain barrier
BC: Breast cancer
BM: Brain metastasis
BoM: Bone metastases
CNS: Central nervous system
CS: Chamber slide
ctrl: control
DCE-MRI: Dynamic contrast-enhanced MRI
DTX: Docetaxel
EC: Endothelial cell
EM: Electron microscopy
ER: Estrogen receptor
exp.: experimental
HBMEC: Human brain microvascular EC
HR: Hormone receptor
ICC: Immunocytochemistry
IHC: Immunohistochemistry
mBC: metastatic BC
MBMEC: Mouse brain microvascular EC
ms: mouse
NVU: Neurovascular unit
PR: Progesterone receptor
qPCR: quantitative polymerase chain reaction
rt.: rat
TC: Tumor cell
TEER: Transendothelial electrical resistance
TN: Triple negative
u: unknown
WB: Western blot
