# Supplementary figures and images for: Impact of Docetaxel on blood-brain barrier function and formation of breast cancer brain metastases
Source: J Exp Clin Cancer Res. 2019 Oct 29;38:434. doi: 10.1186/s13046-019-1427-1 (PMC6819416; doi:10.1186/s13046-019-1427-1)

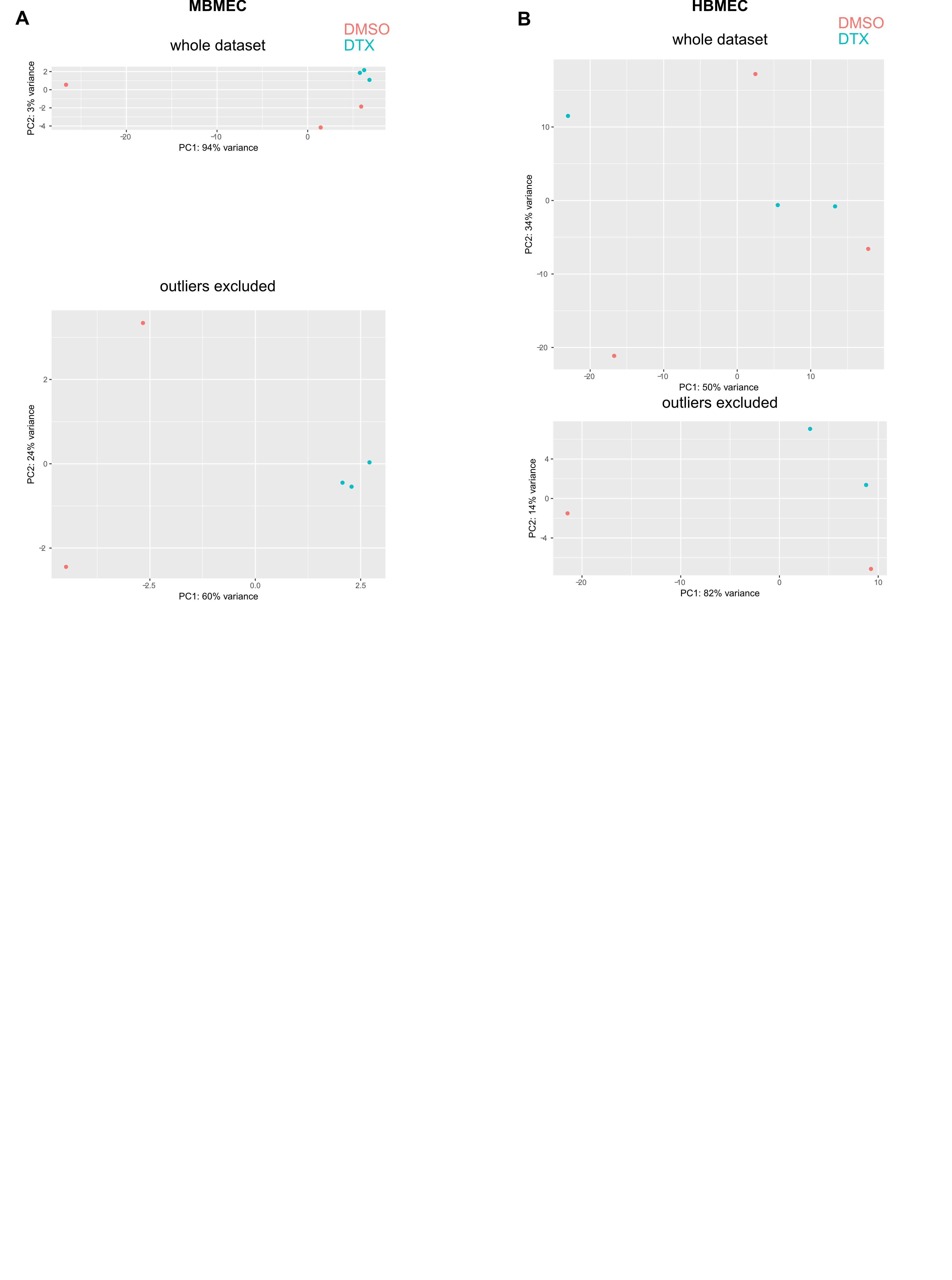

Supplement: Supplementary file 1 — Additional file 1: Figure S1. Principal component analysis (PCA) of RNA-Sequencing data. [file 13046_2019_1427_MOESM1_ESM.tiff]

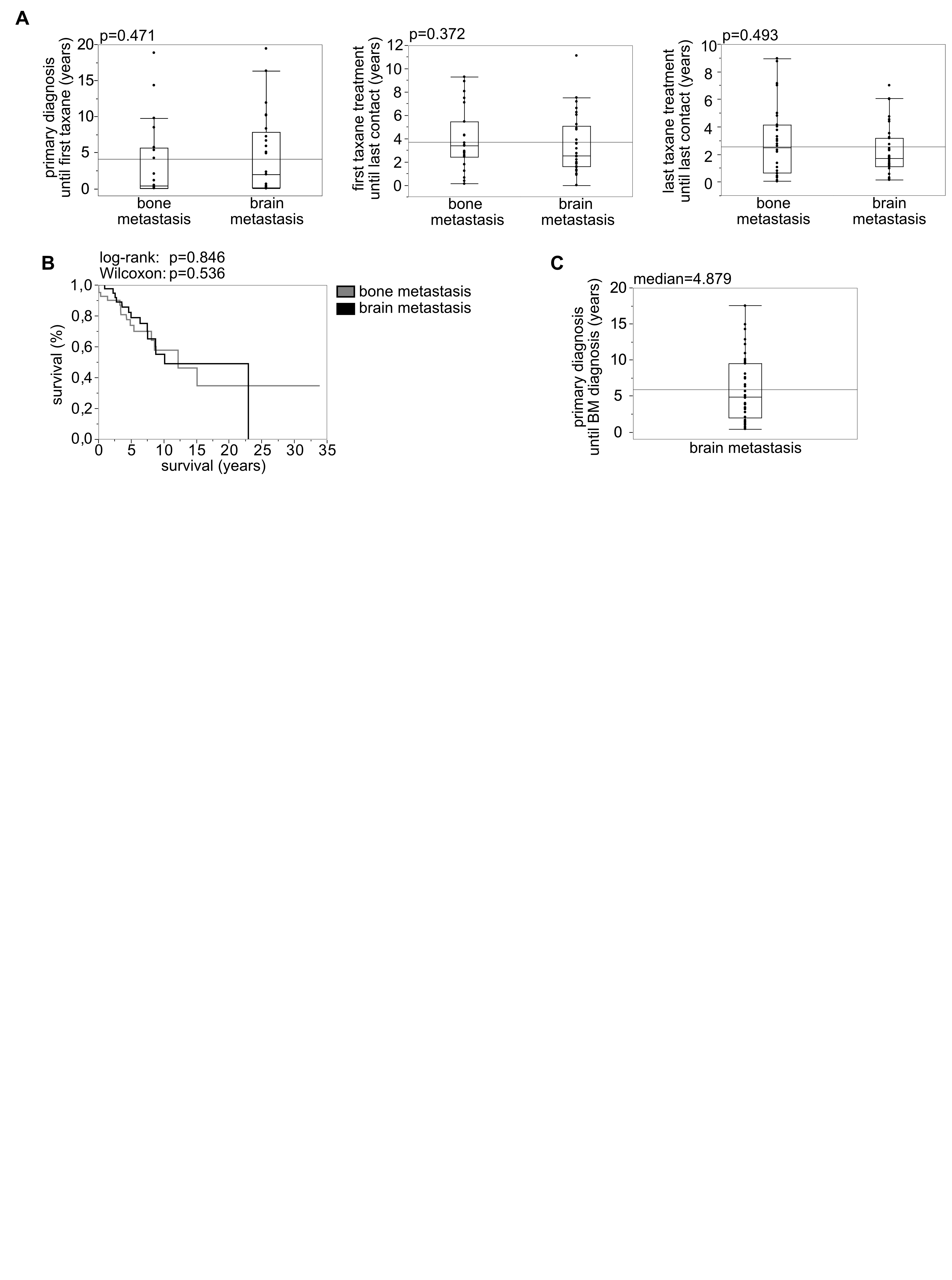

Supplement: Supplementary file 2 — Additional file 2: Figure S2. Start/end of taxane-treatment, follow-up and survival did not differ between BM/ BoM patients. (A) Non-parametric multiple comparisons for each pair using Wilcoxon-method: Years: primary diagnosis until first taxane (N (nBM) = 23, N (BM) = 27); Follow up “first taxane” (N (nBM) = 23, N (BM) = 28); Follow up “last taxane” (N (nBM) = 29, N (BM) = 34). (B) Kaplan-Meier survival curves of BM vs. BoM patients. Curves were compared by log-rank and Wilcoxon tests. (C) Years: primary diagnosis until BM (N (BM) = 38). [file 13046_2019_1427_MOESM2_ESM.tiff]
