# Supplementary material for: Impact of Docetaxel on blood-brain barrier function and formation of breast cancer brain metastases
Source: J Exp Clin Cancer Res. 2019 Oct 29;38:434. doi: 10.1186/s13046-019-1427-1 (PMC6819416; doi:10.1186/s13046-019-1427-1)
Supplement: Supplementary file 3 — Additional file 3: Table S1. HBMEC Patient characteristics. [file 13046_2019_1427_MOESM3_ESM.docx]

**Table S1:** HBMEC Patient characteristics

|  | BR 1 | BR 2 | BR 3 |
| --- | --- | --- | --- |
| patient age/ sex | 29/ m | 51/ m | 23/ f |
| diagnosis | Temporal lobe epilepsy | Temporal lobe epilepsy, Hippocampal sclerosis | Temporal lobe epilepsy, Hippocampal sclerosis |
| operation | Craniotomy, temporal pole resection | Craniotomy, temporal pole resection | Craniotomy, temporal pole resection |
| secondary diagnosis | Nicotine abuse (10py) | Perinatal hypoxic brain injury | prior meningitis |
| epileptic medication | Valproat, Levetiracetam, Oxcarbazepin | Gabapentin, Carbamazepin, Valproat,  Phenytoin, Primidon, Topiramat, Levetiracetam, Oxcarabazepin | Phenytoin,  Valproat, Levetiracetam, Oxcarbazepin |
| additional neuropathologic findings | Focal astrogliosis,  Focal few IDH1R132H-positive cells & few proliferating cells: neuroepithelial tumor cells, no solid tumor formation | No sign of neoplasm | No sign of  neoplasm |

Tissue from three individual patients suffering from epilepsy was used for HBMEC generation. Detailed information about clinically relevant patient characteristics is depicted.
