# Supplementary material for: Impact of Docetaxel on blood-brain barrier function and formation of breast cancer brain metastases
Source: J Exp Clin Cancer Res. 2019 Oct 29;38:434. doi: 10.1186/s13046-019-1427-1 (PMC6819416; doi:10.1186/s13046-019-1427-1)
Supplement: Supplementary file 4 — Additional file 4: Table S2. Primers used for qPCR analysis. [file 13046_2019_1427_MOESM4_ESM.docx]

**Table S2:** Primers used for qPCR analysis

| primers | sequence 5‘-3‘ antisense | sequence 5‘-3‘ sense |
| --- | --- | --- |
| PGP | gctatcacggccaacatctcc | tgtccaacactgaatgctccaa |
| Cldn-5 | tgtcgtgcgtggtgcagagt | tgctacccgtgccttaactgg |
| VE-Cadherin | gcccagccctacgaacctaaa | gggtgaagttgctgtcctcgt |
| Ang2 | acctcgctggtgaagagtcca | tcctgagagcatctgggaaca |
| ABCC1 | atcttgcgcttcccactcaac | gacctccgctcaatgctgtct |
| ABCG2 | tgaaggccgatgttcctttctt | tggagattcttctgctgtgatg |
| ABCC4 | atttcaccgctttctgggaca | gacgacttgcctgctccaact |
